# The genetic landscape of pancreatic head ductal adenocarcinoma in China and prognosis stratification

**DOI:** 10.1186/s12885-022-09279-9

**Published:** 2022-02-18

**Authors:** Yefan Yang, Ying Ding, Yuxi Gong, Sha Zhao, Mingna Li, Xiao Li, Guoxin Song, Boya Zhai, Jin Liu, Yang Shao, Liuqing Zhu, Jiaohui Pang, Yutong Ma, Qiuxiang Ou, Xue Wu, Zhihong Zhang

**Affiliations:** 1 Department of Pathology, the First Affiliated Hospital of Nanjing Medical University, 300 Guangzhou Road, Nanjing, 210029 Jiangsu Province China; 2 Clinical Medicine Research Institution, the First Affiliated Hospital of Nanjing Medical University, 300 Guangzhou Road, Nanjing, 210029 Jiangsu Province China; 3 Nanjing Geneseeq Technology Inc, Nanjing, 210032 Jiangsu Province China

**Keywords:** Pancreatic cancer, NGS, Genomic landscape, Prognosis prediction

## Abstract

**Background:**

Pancreatic ductal adenocarcinoma (PDAC) is the major subtype of pancreatic cancer and head PDACs show distinct characteristics from body/tail PDACs. With limited studies based on Asian population, the mutational landscape of Asian PDAC remains unclear.

**Methods:**

One hundred fifty-one Chinese patients with head PDAC were selected and underwent targeted 425-gene sequencing. Genomic alterations, tumor mutational burden, and microsatellite instability were analyzed and compared with a TCGA cohort.

**Results:**

The genomic landscape of Chinese and Western head PDAC had identical frequently-mutated genes including *KRAS*, *TP53*, *SMAD4*, and *CDKN2A*. *KRAS* hotspot in both cohorts was codon 12 but Chinese PDACs containing more G12V but fewer G12R variants. Potentially pathogenic fusions, *CHD2-BRAF* and *KANK1-MET* were identified in two *KRAS* wild-type patients. Serum cancer antigens CA125 and CA19-9 were positively associated with *SMAD4* alterations while high CEA was enriched in wild-type *CDKN2A* subgroup. The probability of vascular invasion was lower in patients with *RNF43* alterations. The nomogram developed including histology grade, the mutation status of *SMAD4, TGFBR2,* and *PREX2* could calculate the risk score of prognoses validated by Chinese and TCGA cohort.

**Conclusions:**

Chinese head PDAC contained more *KRAS* G12V mutation than Western population. The well-performed nomogram may improve post-operation care in real-world practice.

**Supplementary Information:**

The online version contains supplementary material available at 10.1186/s12885-022-09279-9.

## Background

Pancreatic cancer is one of the most deadly cancers with a five-year survival rate of less than 9% [1]. The prognosis of pancreatic patients is highly associated with the diagnosis stage. For early-stage patients, the five-year survival rate can reach 37% but for those with distant metastases, it drops to 3%. The incidence rate of pancreatic cancer is trending upward in China and worldwide [1, 2]. Pancreatic ductal adenocarcinoma (PDAC) is the leading histological subtype and covers over 90% of all pancreatic cancers [3]. 80% of PDACs are located at the head of the pancreas, which arises from different embryonic origins compared with the tail of the pancreas. The ventral bud forms the posterior part of the head or uncinate process, while the dorsal bud forms the rest of the pancreas [4]. The prognosis between head PDAC and body/tail PDAC were extremely controversial. Some studies indicated primary head PDAC has a better prognosis than body/tail PDAC [5, 6], while others suggested the opposite outcome in resectable or early advanced PDAC [7, 8]. As the comprehensive studies revealed 4 molecular subtypes of PDAC, squamous, pancreatic progenitor, immunogenic, and aberrantly differentiated endocrine exocrine (ADEX), the squamous subtype was associated with a poor prognosis [9, 10]. Later studies found head and body/tail PDAC have different gene expression signatures and are rich in different molecular subtypes, with body/tail PDAC containing more squamous subtype and less immunogenic subtype than head tumors, which may contribute to their diverse clinical manifestations and outcomes [11, 12].

Previous whole-genome studies have investigated the mutational profiles of PDAC in Western populations and uncovered four abundantly common mutations, including *KRAS, TP53, SMAD4*, and *CDKN2A *[13, 14]. Due to the limited studies based on the Asian population, the genetic landscape of Asian PDAC patients remains unclear. Several genes were found survival-related in PDAC [15, 16]. However, no risk stratification was developed based on intra-tumor genetic heterogeneity.

The incidence of mismatch repair deficiency in PDAC is only 2% according to a Danish cohort study [17]. But it’s still worth investigating further as they may benefit from immune therapy especially under the circumstance of very limited treatment options for PDAC patients. Tumor biomarker CA19-9 is applied in clinical practice for PDAC diagnosis, treatment guidance, and follow-ups [18]. Serum tumor marker carcinoembryonic antigen (CEA) and carbohydrate antigen CA125 are widely used in colorectal can ovarian cancer screening. Over the past few years, it is revealed they may benefit PDAC diagnosis and postoperative monitor [19, 20]. However, the correlation between these antigen levels and genomic alterations is rarely studied. To comprehensively study the genetic alterations in the Chinese PDAC patients and if these alterations affect the clinical outcome, 151 Chinese PDAC patients were retrospectively investigated and a nomogram was established to calculate postoperative risk score to predict prognosis.

## Methods

### Patient cohort and samples

A total of 153 patients diagnosed with head PDAC and underwent surgery (both resectable and palliative) in the First Affiliated Hospital of Nanjing Medical University (also known as Jiangsu Province Hospital), China between October 2017 to February 2019 were retrospectively assessed in this study in accordance with the Declaration of Helsinki. The study was approved by the ethics committee of the First Affiliated Hospital of Nanjing Medical University(2020-SR-273), and informedconsents were obtained from all participants. Patients who received neoadjuvant chemotherapy or died of post-operation complications within 30 days after surgery were excluded. Formalin-fixed, paraffin-embedded (FFPE) tumor samples were obtained from all patients for DNA extraction and sequencing. Two patients were excluded from the following analysis whose samples failed the quality control process.

The western cohort that consisted of 91 head PDAC patients was identified from The Cancer Genome Atlas dataset (version # 2016–01-28). Whole-exome sequencing data of the TCGA cohort were analyzed.

### DNA extraction, library preparation, and targeted sequencing

Genomic DNA was extracted from FFPE specimen using QIAamp DNA FFPE Tissue Kit (Qiagen), according to the manufacturers’ protocols. DNA was quantified using the dsDNA HS Assay Kit on a Qubit 3.0 Fluorometer (Life Technologies, Carlsbad, CA). The complete DNA concentrations are listed in Supplementary Table S5 with a median concentration of 116 ng/μL (range: 9.3—368 ng/μL). The extracted DNA was also qualified using a Nanodrop2000 (Thermo Fisher Scientific, Waltham, MA) and the same amount of DNA (2000 ng) was uploaded for the following sequencing process. For the five samples whose total amount of extracted DNA was below 2000 ng, all extracted DNA was used (837—1890 ng) and all had passed the following quality control. Sequencing libraries were prepared using the KAPA Hyper Prep Kit (KAPA Biosystems). Genomic DNA was sheared into 200–350 bp fragments using the Covaris M220 instrument (Covaris) and underwent end-repairing, A-tailing, and ligation with indexed sequencing adapters sequentially. Libraries were then amplified by PCR and purified using Agencourt AMPure XP beads. For targeted enrichment, DNA libraries were pooled for hybridization using customized xGen lockdown probes (Integrated DNA Technologies) for 425 cancer-related genes. Captured libraries were subjected to PCR amplification with KAPA HiFi HotStart ReadyMix (KAPA Biosystems). The purified library was quantified using the KAPA Library Quantification Kit (KAPA Biosystems), and its fragment size distribution was analyzed using a Bioanalyzer 2100. Enriched libraries were amplified and subjected for next-generation sequencing (NGS) on Illumina Hiseq4000 platforms (Illumina) using paired-end sequencing to a targeted mean coverage depth of 700 × , which was controlled by data collection software (Illumina).

### Sequencing data processing

FASTQ files were processed with Trimmomatic for quality control. Sequencing data were mapped to the Human Genome version 19 (hg19) using the Burrows-Wheeler Aligner [21]. PCR duplicates were removed by Picard (available at https://broadinstitute.github.io/picard/) and the Genome Analysis Toolkit (GATK) was used to perform local realignments around indels and base quality recalibration [22]. Single nucleotide variants and indels were called by VarScan2 and HaplotypeCaller / UnifiedGenotyper in GATK, with the mutant allele frequency (MAF) cutoff as 0.5% and a minimum of three unique mutant reads. Common SNPs were removed using dbSNP and the 1000 Genome project [23]. The resulting somatic variants were further filtered through an in-house list of recurrent sequencing errors that were generated from over 10,000 normal control samples on the same sequencing platform.

Copy number variations (CNVs) were called as losses or gains relative to the overall sample-wide estimated ploidy as previously described [24, 25]. Arm gain or loss was called when more than 50% of the chromosome have copy number gain or loss. Gene fusion (common fusion regions/introns captured in the target panel) was called using DELLY [26]. Tumor mutational burden (TMB) was defined as the number of somatic synonymous mutations per megabase in each sample, with hotspot/fusion mutations excluded.

Fifty-two microsatellite loci are incidentally captured and evaluated during the targeted 425 gene panels. Based on previous validation studies, a fraction of > 0.4 (> 40% unstable loci) was considered microsatellite unstable [27, 28]. Structural variants were detected using FACTERA (Fusion And Chromosomal Translocation Enumeration and Recovery Algorithm) with the default parameter [29]. Likely germline mutations were identified using a computational prediction method, namely Toseq (Genseeq Technology), which is an algorithm developed using machine learning based on past archived patients’ mutational features.

### Data collection and analysis

Clinical pathological features including age, sex, tumor diametre, microscopic vascular and perineural invasion, pTNM stage, resection margin, family, and personal cancer history were collected in this study. Resection margin status was classified into R2 (macroscopically positive), R1 (macroscopically negative but tumor found within less than 1 mm from the margin under the microscope), and R0 (macroscopically and microscopically negative). *AJCC staging manual 7*^*th*^* edition* was used to normalize tumor stage in Chinese cohort with the TCGA cohort. Tumor stage in other parts of this article was under the guidance of *AJCC staging manual 8*^*th*^* edition*.

### Statistical analysis and nomogram development

Data were analyzed using R 4.0.1 [30]. Categorical variables between groups were compared using χ^2^ or Fisher’s exact test. Continuous variables between groups were compared using two-sided Mann–Whitney U test should the variables failed to obey normal distribution and using Student’s t-test if they follow normal distribution. Kaplan–Meier method was used to determine median overall survival (OS) and the significance of survival analysis was determined by the log-rank test. A nomogram was developed to predict one-year survival post-operation based on multivariate Cox regression using R package “rms”. Variables with a *P* value of less than 0.1 in univariable analysis and met proportional hazard assumption were chosen for multivariable Cox regression. Patients in Chinese cohort were assigned in chronological order to two groups, training group and Chinese external validation group for nomogram validation, which contains 92 and 49 patients, respectively. 65 patients in the western cohort with available CNV data comprised the second validation cohort. X-tile was used to determine the cut-off of the risk score calculated by the nomogram [31]. Other R packages used in this study include “ComplexHeatmap”, “ggplot2”, “survival”, “survminer”, “waterfall”, and “Hmisc”. Stage IV cases were excluded in survival analysis. *P* < 0.05 was considered statistically significant. 

## Results

### Clinical and pathological features

A total of 151 Chinese head PDAC patients were enrolled in this study. The median age at diagnosis was 63 (ranging from 31 to 85) and 50.3% (76/151) were male (Table 1). The majority (80.1%, 121/151) of patients have stage I or II PDAC. 111 patients had lymph nodes metastasis, including regional (105/151, 69.5%), non-regional (3/151, 2.0%), and both (3/151, 2.0%) lymph nodes metastasis. Other pathology stages, histology grade, and invasion status were summarized in Table 1. Additionally, 4 out of 151 (2.6%) patients had solitary liver metastasis and thirteen (8.6%) patients reported cancer history including colorectal (4/13), breast (3/13), endometrial (2/13), bladder(2/13), esophageal (1/13), gastric (1/13), and basal cell skin cancer (1/13). In comparison, the TCGA (*n* = 91) had a higher proportion of early-stage patients (stage I/II: 95.6%, 87/91). The pathology T stage and histologic grade were significantly higher in Chinese cohort, while N stage was higher in TCGA cohort. More patients in the TCGA cohort achieved microscopically margin-negative resection (53.8% vs. 33.1%). The invasion information was not available from TCGA database.

**Table 1:** Clinical characteristics of this study and TCGA cohorts

Characteristics	This study			TCGA	*P-*value
	**All (*N* = 151)**	**Training (*N* = 92)**	**Validation (*N* = 49)**	**All (*N* = 91)**	
Age(years)					
< 65	86(57.0%)	55(59.8%)	26(53.1%)	41(45.1%)	0.08
≥ 65	65(43.0%)	37(40.2%)	23(46.9%)	50(54.9%)	
Gender					
Male	76(50.3%)	49(53.3%)	22(44.9%)	49(53.8%)	0.69
Female	75(49.7%)	43(46.7%)	27(55.1%)	42(46.2%)	
Stage (AJCC 7^th^)					
I-II	121(80.1%)	82(89.1%)	39(79.6%)	87(95.6%)	< 0.01
III	22(14.6%)	10(10.9%)	12(24.5%)	3(3.3%)	
IV	8(5.3%)	0(0%)^a^	0(0%)^a^	1(1.1%)	
Pathology T stage					
T1-2	5(3.3%)	2(2.2%)	3(6.1%)	11(12.1%)	0.01
T3-4	146(96.7%)	90(97.8%)	46(93.9%)	80(87.9%)	
Pathology N stage					
N0	42(27.8%)	27(29.3%)	15(30.6%)	14(15.4%)	0.04
N1-2	109(72.2%)	65(70.7%)	34(69.4%)	77(84.6%)	
Neoplasm histologic grade					
G1-2	120(79.5%)	84(91.3%)	31(63.3%)	47(51.6%)	8.01E-6
G3	31(20.5%)	8(8.7)	18(36.7%)	44(48.4%)	
Surgical margin resection status					
R0	50(33.1%)	28(30.4%)	18(36.7%)	49(53.8%)	1.36E-5
R1 + R2	99 + 2(66.9%)	64(69.6%)	31(63.3%)	37(40.7%)	
Rx/NA	0(%)	0(%)	0(%)	5(5.5%)	
Vascular invasion					
Negative	86(57.0%)	55(59.8%)	27(55.1%)	-	
Positive	65(43%)	37(40.2%)	22(44.9%)	-	
Perineural invasion					
Negative	14(9.3%)	4(4.3%)	7(14.3%)	-	
Positive	137(90.7%)	88(95.7%)	42(85.7%)	-	

^a^10 stage IV patients were excluded from prediction modeling

### Genetic landscape of Chinese cohort and comparison with TCGA cohort

**Fig. 1:**
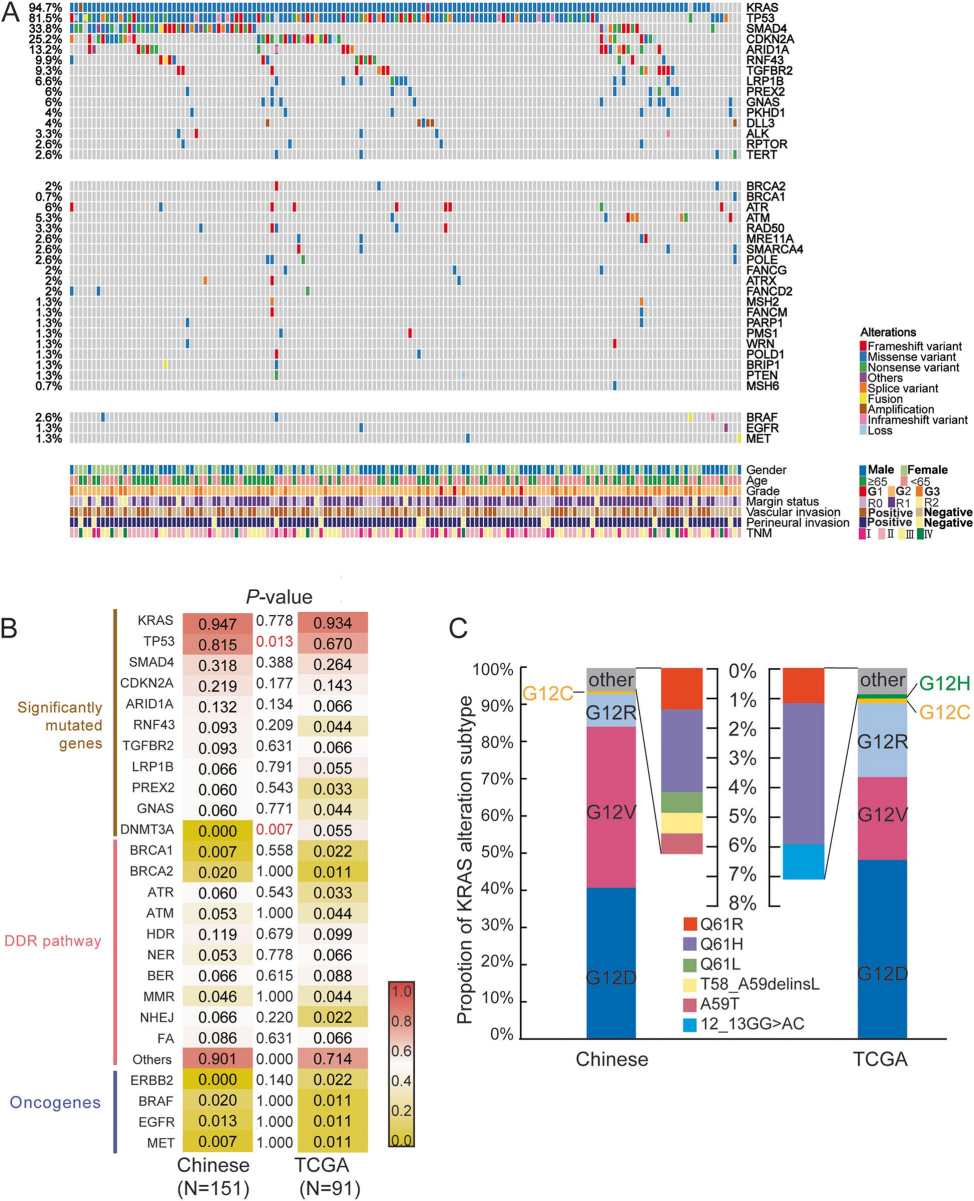
Concurrent mutations in Chinese cohort and incidence comparison with TCGA cohort. **A** The oncoprint of all patients in the Chinese head PDAC cohort. The top panel includes the most frequently mutated genes. The genes in the middle and the bottom panel are DNA damage repair pathway related and other oncogenic genes, respectively. The alteration frequency of each gene is labled on the left side. **B** The alteration frequency comparison between the cohort in this study and the TCGA cohort with a coloured scale. The *P-*value less than 0.05 is statistically significant and highlighted in red. **C** A bar plot shows the proportion of each KRAS alteration subtype in this study and the TCGA cohort

The genomic mutation profiles of all 151 patients in Chinese cohort were generated by the panel NGS targeting 425 cancer-related genes. The median TMB was 5.7 Mutations/Mb (ranging from 0 to 73.6). As shown in Fig. 1A, the most frequently mutated genes were *KRAS* (94.7%), *TP53* (81.5%), *SMAD4* (33.8%), and *CDKN2A* (25.2%) which were also at the top in TCGA cohort suggesting a similar mutational pattern in Chinese and Western populations. By comparing the frequencies of commonly mutated genes, DNA damage repair pathway, and other oncogenes between this study and TCGA cohort, the majority of them showed roughly equal frequency such as *TGFBR2* (9.3% vs. 6.6%, *P*-value 0.63) and *PREX2* (6.0% vs. 3.3%, *P*-value 0.54)*.* However, Chinese cohort tended to have more *TP53* mutations (81.5% vs. 67.0%) but no *DNMT3A* alteration (0% vs. 5.5%), the *P-*value of which were 0.013 and 0.007, respectively (Fig. 1B).

The correlations between clinicopathological features and genomic mutations were investigated in the Chinses cohort. Serum tumor marker CEA and carbohydrate antigens, CA125 and CA19-9, were measured preoperatively. CA19-9 level didn’t correlate to tumor stage in the Chinese cohort (*P* = 0.85). A strong correlation between CEA and *CDKN2A* was observed as CEA level was significantly higher in patients with wild-type *CDKN2A* (Supplementary Figure S2A, mean 8.37 vs. 4.10, *P* = 0.038). Furthermore, both preoperative CA125 and CA19-9 levels were positively associated with *SMAD4* alterations (*P* = 0.009 & 0.008, respectively*,* Supplementary Figure S2B-C), which were enriched in older patients(> 50-year-old) (Supplementary Figure S2D, *P* = 0.031).

As vascular invasion occurred in nearly half cases (65/151, 43.0%) of Chinese cohort, the association between genomic mutations and vascular invasion were investigated. As shown in Supplementary Figure S3, *RNF43* alteration was associated with negative microscopic vascular invasion, the odds ratio of which was 0.18 (95%CI: 0.02–0.84, *P-*value 0.01).

*GNAS* mutations were found in all three colloid carcinomas and six conventional PDACs. All three colloid carcinomas and six conventional PDAC have intraductal pancreatic neoplasm(IPMN) adjacent to invasive carcinoma, which was identified in 20(20/151, 13%) cases. Most *GNAS* mutations(8/9, 89%) were located in codon 201 (R201C, R201H & R201L). 8 patients harboring *GNAS* alteration also had *KRAS* mutation. The one without concurrent *KRAS* mutation had a histological appearance of colloid carcinoma, with somatic *ATM* and *APC* mutation. TCGA cohort showed co-occurrent *GNAS* and *RNF43* mutations(*P* = 0.024), but such a relationship was not found in Chinese cohort(*P* = 0.20). Survival analysis showed no difference between *GNAS*/*RNF43* altered patients and wild-type patients. *RICTOR*, *PREX2*, *TGFBR2* mutations were associated with IPMN-associated PDAC(*P* < 0.05). No correlations were found between other histological subtypes and gene alterations.

### KRAS mutation in Chinese and TCGA cohort

As *KRAS* was mutated in over 90% of patients in both Chinese and TCGA cohort, the alteration subtypes of *KRAS* mutations were futher investigated. As shown in Fig. 1C, the hotspot of *KRAS* is codon 12 which contributed to 93.8% and 92.9% of all *KRAS* mutations in this study and TCGA cohort, respectively. The proportion of *KRAS* G12D in the two cohorts was similar (this study 40.7% vs. TCGA 48.2%) while Chinese cohort had a higher mutation rate of G12V (43.5% vs. 22.4%) but lower with G12R (9.0% vs. 20.0%) comparing to TCGA. The TCGA cohort also had a unique subtype, G12H(1/91), which wasn’t detected in any patients from Chinese cohort. These G12 mutational subtypes were found not to be associated with OS as analyzed in this study (Supplementary Figure S1A). To be noted, ten stage IV patients were excluded from all survival analyses. Other *KRAS* subtypes mainly occurred in codon 59 and 61, including single or multiple amino acid substitution, with quite low frequency in both cohorts (Fig. 1C). *BRAF* missense mutations were identified in two *KRAS*-mutation patients at low allele frequency, which occurred in *BRAF* V413M and G469A, respectively.

The mutational status of eight patients with wild-type *KRAS* in Chinese cohort were examined and results showed that they all harbored other RAS pathway-related gene alterations including *BRAF, BRCA1/2, EGFR, MET, TP53, TSC1,* and *TYMS.*

### Structural variances in Chinese head PDAC cohort

In Chinese cohort, a total of 35 CNVs were detected in 22 patients involving 19 genes and two-thirds (23/35) were amplification (Fig. 1A). The most frequently amplified gene was *DLL3* (*n* = 5) followed by *AKT2* (*n* = 3), while *CDKN2A* (*n* = 4), *CDKN2B* (*n* = 3)*,* and *SMAD4* (*n* = 2) incurred copy number loss. Furthermore, 14 patients were detected with gene rearrangements (*n* = 20). It’s worth noting that two *KRAS* wild-type patients (P74 and P75) harbored potential pathogenic fusions. P74 had a *CHD2-BRAF* rearrangement which maintained the intact kinase domain of *BRAF* encoded by exon 11 to 18 (Supplementary Figure S4A). No gene mutations but a *MET* gene rearrangement was found in P75 by the targeted panel NGS, where the intact *MET* kinase domain was fused to the coiled-coils of *KANK1* (Supplementary Figure S4B).

### Mismatch repair (MMR) deficiency (d-MMR) and microsatellite instability (MSI) events

The reported frequencies of d-MMR in PDAC varied greatly and here in the present study nine patients (9/151, 6%) were found with somatic and/or germline MMR mutations, including *MLH1*, *MSH2*, *MSH6*, *PMS1*, and *POLD1* (Table 2). Only one *MSH2* splice mutation was reported likely pathogenic in the database. Others have uncertain significance or weren’t recorded. Among the four patients with germline MMR mutations, two had MSI which was also detected in a third patient (P124) harboring somatic *MSH2* mutation and accompanied by high TMB. The three MSI patients in Chinese cohort displayed a better prognosis as no one died of PDAC in 12 to 29 months follow-up comparing to a median survival of 18 months in microsatellite stable patients. However, survival analysis found no significant association between MSI and prolonged survival (Supplementary Figure S1B, *P* = 0.14). Only four patients (4/151, 2.6%) were reported with a TMB over 20 mutations/Mb which were all identified as d-MMR and three of them harbored germline mutations. The remaining one was the above-mentioned P74. Statistical analysis found no correlations between high TMB and prolonged OS (*P-*value = 0.43, Supplementary Figure S1C). Meanwhile, all four germline d-MMR patients had previous cancer history and/or first-degree relatives’ cancer history (Table 2).

**Table 2:** Nine d-MMR patients’ medical histories and mutational profiles

Case	Sex	Age	MMR gene mutation	Medical history (age)	Family medical history	MSI	TMB (muts/Mb)
17	F	66	MSH6(p.R911Q)	Breast (54)	-	No	9.2
22	M	41	PMS1(p.L146Ffs*5)	-	-	No	8
42	M	55	POLD1(p.P116Hfs*53) MLH1(Germline: splice donor)	Bladder (41); Colon (54)	Mother: unknown cancer	Yes	73.6
51	M	55	MSH6(Germline: p.R248Tfs*8)	-	Father: colon; Mother: esophagus	No	23
63	F	54	MSH2(Germline: p.H839R)	-	Father: bile duct	No	5.7
83	M	54	PMS1(p.L813R)	-	-	No	8
99	F	55	MSH2(splice), MSH2(Germline: p.A714Lfs*6)	Endometrial (49)	-	Yes	23
102	F	66	POLD1(splice)	-	-	No	3.4
124	F	74	MSH2(ex7_6del)	-	-	Yes	59.8

*F* Female, *M* Male, *MMR* mismatch repair, *MSI* microsatellite instability, *TMB* tumor mutational burden

### Nomogram calculating risk score and predicting prognosis

**Fig. 2:**
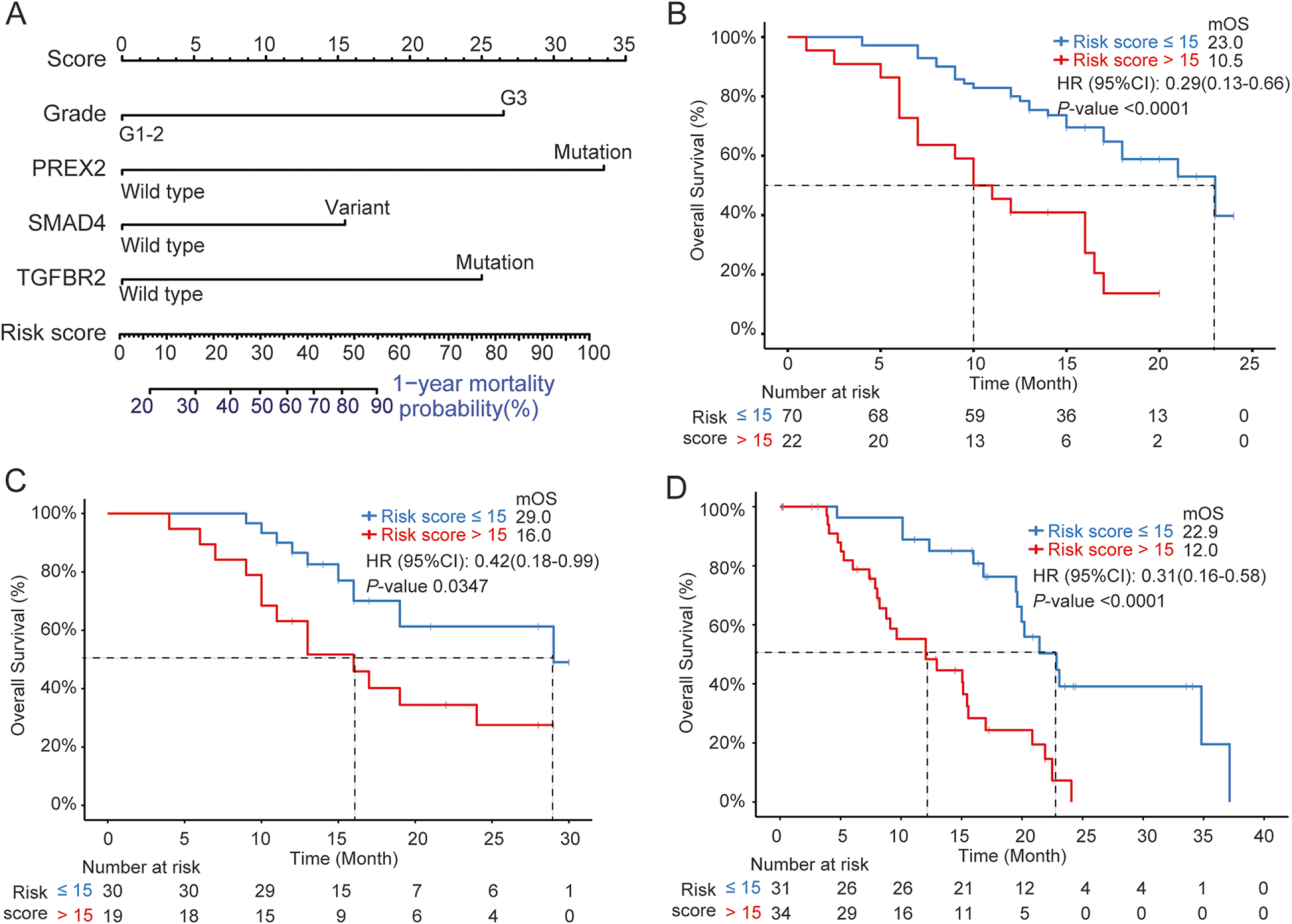
Nomogram for risk score calculation and its performance validation. **A** The nomogram calculating the risk score of one-year mortality probability based on the selected four features. The status of each feature corresponds to the score on the top panel. The risk score is the sum of the scores corresponding to each feature which then represents the one-year mortality probability according to the scale bars. The risk score of 15 is the cutoff of the high and low risk groups. The overall survival (OS) curves of patients with high (> 15, red) and low (≤ 15, blue) in the Chinese training cohort (**B**), Chinese validation cohort (**C**), and the TCGA cohort (**D**) are shown. Median OS (MOS), HR (95% CI), and P-value are labeled on the right-up corner of each figure

To establish a model to predict the prognosis of PDAC patients, Chinese cohort was divided into training (*n* = 92) and validation (*n* = 49) cohorts after excluding 10 stage IV patients. The clinical characteristic distribution of the two sub-cohorts remained comparative (Table 1). Univariate and multivariate analyses were performed on training group to evaluate the association between all factors and OS. As shown in Table 3, the four factors with a *P-*value of less than 0.1 in the univariate analysis were included in the multivariate analysis: histology grade, *SMAD4, TGFBR2,* and *PREX2* mutations (Fig. 2A). A risk score was calculated based on the nomogram and the cut-off of 15 was determined by X-tile [31]. Patients with a risk score of over 15 were considered as high risk whose one-year mortality probability was over 28%. The median OS of low-risk patients was 23.0 months while for the high-risk group, it dropped to 10.5 months (Fig. 2B). The performance of the nomogram was then assessed in the Chinese validation cohort and TCGA validation cohort. As shown in Fig. 2C, the median OS of low-risk and high-risk patients in the Chinese validation cohort was 29.0 and 16.0 months, respectively with a *P-*value of 0.0347. Similarly, in the TCGA validaton cohort, which included PDAC in all sites, the nomogram was able to accurately predict the prognosis with a 0.31 HR (95% CI: 0.16–0.58, Fig. 2D).

**Table 3:** Univariate and Multivariate analysis of patients’ characteristics and OS

	**Univariate analysis**			**Multivariate analysis**		
	HR	95% CI	*P-*value	HR	95% CI	*P-*value
Gender: Male (vs. Female)	1.22	0.67 ~ 2.24	0.52	-	-	-
TNM Stage: III (vs. I-II)	1.07	0.56 ~ 2.06	0.84	-	-	-
Margin status: R1-2 (vs. R0)	1.55	0.76 ~ 3.14	0.22	-	-	-
Vascular Invasion: Positive (vs. Negative)	1.44	0.79 ~ 2.64	0.23	-	-	-
Perineural Invasion: Positive (vs. Negative)	2.63E + 07	0 ~ Inf	0.13	-	-	-
pT: T3-4(vs. T1-2)	1.49	0.81 ~ 2.76	0.2	-	-	-
pN: N1-2(vs. N0)	1.62	0.80 ~ 3.30	0.18	-	-	-
KRAS mutation (vs. WT)	0.88	0.21 ~ 3.70	0.863	-	-	-
TP53 mutation (vs. WT)	0.64	0.32 ~ 1.27	0.199	-	-	-
Age: ≥ 65 yrs (vs. < 65 yrs)	2.17	1.19 ~ 3.95	0.010	1.79	0.93 ~ 3.47	0.084
Grade: G3 (vs. G1-2)	2.18	0.85 ~ 5.56	0.095	3.79	1.35 ~ 10.6	0.011
SMAD4 variant (vs. WT)	1.92	1.06 ~ 3.50	0.030	2.05	1.05 ~ 4.01	0.036
TGFBR2 mutation (vs. WT)	3.15	1.44 ~ 6.91	0.002	3.55	1.55 ~ 8.15	0.003
PREX2 mutation (vs. WT)	4.03	1.57 ~ 10.4	0.002	4.03	1.48 ~ 10.98	0.006
ATM mutation (vs. WT)	2.47	0.88 ~ 6.96	0.077	1.43	0.45 ~ 4.51	0.545
ERCC1 SNP (vs. WT)	0.58	0.31 ~ 1.08	0.081	0.54	0.28 ~ 1.04	0.067

*WT* wild-type, *HR* hazard ratio, *CI* confidence interval, *Inf* infinity

## Discussion

In the present study, a gene-related nomogram was developed to predict 1-year postoperative risk and investigated the genetic landscape of head PDAC based on the Chinese population. Nomogram was internally and externally validated and shows good performance in PDAC at all sites and races. It’s worth pointing out that different technologies were used in the two cohorts for DNA sequencing. Chinese cohort used extracted DNA from FFPE tumor samples which then underwent a panel NGS targeting 425 cancer-related genes. In contrast, the TCGA cohort used fresh-frozen samples and WES for mutational analysis. Other than that, the average examined lymph node number in the TCGA cohort is higher than Chinese cohort (19 vs. 16) which may explain the overall lower pathology N stage observed in Chinese cohort. A higher *TP53* mutational frequency (81.5%) was observed in Chinese cohort than the TCGA cohort (67.0%) which is also higher than another pan-site PDAC study (73.5%) reported by Singhi et al. [32]. Besides, more high-TMB (> 20 mutations/Mb) patients were present in Chinese cohort (2.6%) comparing to previous studies, in which the reported TMB-high rates were less than 1% [32, 33]. This might be caused by the relatively high incidence of d-MMR in this study (9%) as other studies showed about 1% d-MMR by NGS and IHC [34]. Furthermore, whether MSI is associated with survival remained inconclusive and no significant association was found between MSI and prolonged survival in Chinese cohort [35]. However, three MSI patients did display a better prognosis who were all alive at the latest follow-up in the period of 12, 19, and 29 months compared to the median OS of 18 months in microsatellite stable patients. And these MSI patients were all TMB-high, which is consistent with previous observations [36].

Studies have found that somatic *GNAS* and *RNF43* mutations were recurrently identified in IPMN [37, 38], so the relationship between *GNAS*/*RNF43* mutations and PDAC with adjuvant IPMN was investigated. Majority of the *GNAS* mutations occurred in codon 201, which is consistent with the previous report [38]. Studies had shown colloid carcinomas of the pancreas were arose in association with IPMN, and GNAS codon 201 mutations can be identified in the majority of colloid carcinoma [39, 40], which are also verified in present study. *RNF43* alteration wasn’t related to any histological subtypes or co-altered with *GNAS*, however, alterations of *RNF43* may contribute to negative vascular invasion. In human hepatocellular carcinoma (HHC), *RNF43* overexpression frequently occurred and study had shown correlated with *RNF43* expression and vascular invasion [41].

PDAC is a tumor driven by *KRAS* mutation, which explained its striking prevalence of over 94% [42]. Studies have shown *KRAS* downstream signaling is affected by different *KRAS* mutations in an allele-specific manner [43, 44]. Therefore, personalized therapies according to specific *KRAS* mutations are being extensively investigated. Several inhibitors have been developed targeting *KRAS* G12C mutation to inhibit *KRAS* signaling, which unfortunately is quite rare in PDAC, and not present in Chinese cohort [45, 46]. Other inhibitors targeting *KRAS* G12V, G12D, or G12A were currently under pre-clinical development and showed promising results in pancreatic patient-derived cell lines and xenografts [47]. The present study revealed a difference in Chinese and Western *KRAS* mutation spectrum. Chinese PDAC had a higher mutation rate of *KRAS* G12V but a lower *KRAS* G12R mutation rate comparing to TCGA cohort. Two recent studies presented at the 2020 ESMO Congress about Chinese PDAC molecular profiling also reported a similar *KRAS* G12 spectrum as this study [48, 49].

Our study showed Chinese cohort had more *TP53* mutation than TCGA cohort. The mutation frequency of *TP53* varies from study to study but is usually over 50%. Sinn et al.reported *TP53* mutations in 60% of the 368 PDAC patients enrolled in Germany and Austria which is comparable to the frequency of TCGA cohort we reported here (67%) [50]. Lin Shui et al.reported more *TP53* mutations in a Chinese PDAC cohort compared to TCGA (62.05% vs 51%) [51]. To be noted, these two studies didn’t specify the subtype of PDAC (head vs body/tail). Another study based on the Chinese population identified 81.8% of patients (*n* = 154) carrying *TP53* mutations [52]. Notably, no significant difference was observed in *TP53* mutation frequency between the 85 head and the 69 body/tail PDAC patients in their study. Thus, the ethnic difference could be a potential explanation of different *TP53* mutation frequencies. The cohort sizes of the above-mentioned Chinese studies were similar to ours which might lead to cohort bias, further larger sample study is needed to validate the results.

Previous studies suggested BRAF V600E mutations and in-frame deletions near the αC-helix region of the kinase domain are mutually exclusive with *KRAS* mutations [13, 53]. However, about 0.3–0.4% of *KRAS* mutant PDACs have concurrent *BRAF* mutations [32, 54], which explained the concurrent *BRAF* and *KRAS* mutations in Chinese cohort. In *KRAS* wild-type PDAC patients, two novel potentially pathogenic fusions, *CHD2* –*BRAF* and *KANK1*-*MET*, were discovered. The former retained an intact BRAF kinase domain, which might cause the activation of *BRAF* signaling. The intact *MET* kinase domain of *KANK1*-*MET* fusion was fused with the coiled-coils of *KANK1*, which were located in the N-terminus of *KANK1* and reported to be required for *KANK1* associated fusion induced cell growth and signaling [55]. It’s worth investigating the efficacy of second-generation *BRAF* inhibitors and *MEK* inhibitors in these patients in the future [56].

This study reported, to our knowledge, for the first time the relationship between common tumor biomarker and gene alterations. Further larger sample analysis needs to be performed to verify the results and adjust for possible involvement of other characteristics. Higher preoperative CA125 and CA19-9 were associated with *SMAD4* alterations, and elevated CEA was associated with *CDKN2A* wild-type. Elevated CA125 and CA19-9 were associated with a worse prognosis in PDAC [57, 58]. *SMAD4* was also found to be survival-related in several studies and this study [15, 59], whether there are common pathways involved in *SMAD4* mutation and elevated tumor biomarkers needs future experiments to clarify. Previous studies suggested CA19-9 levels were correlated to TNM staging as high pre-operation CA19-9 was associated with adverse pathologic features and advanced stage [18], however, no correlation was found in the Chinese cohort. Study has shown as CA19-9 increased, the tumor trends toward unresectable [60]. Since all samples from the cohort were obtained from resected tumors, it is our hypothesize that many high CA19-9 patients were excluded due to the tumor being unresectable, resulting in the irrelevancy. Because of this, the correlation between *SMAD4* and CA19-9 was less likely due to the bias of increased tumor staging.

*SMAD4* alteration was identified as a predictive marker of short OS in this study. However, the association of *SMAD4* and onset age was controversial in previous studies. The study showed that *SMAD4* alterations were more enriched in older patients which is consistent with a large-size (*n* = 3,594) study which suggested patients older than 50 were more likely to harbor *SMAD4* alterations [32]. However, Ben-Aharon et al*.* reported a higher mutational rate of *SMAD4* in younger patients with a cutoff of 55 years old [61]. Checking the patients' characteristics between the studies, we found the majority of early-onset patients (80%) in Ben-Aharon et al*.*’s cohort were of stage IV, which may affect *SMAD4* mutation prevalence as loss of *SMAD4* can lead to tumor metastases [62, 63].

The two gene alterations, *SMAD4* and *TGFBR2*, included in the established nomogram are partners in the TGF-β signaling pathway [64]. A recent study suggested the predominant function of *SMAD4* in collective invasion in PDAC organoids and somatic mutation of *TGFBR2* also showed a similar invasion phenotype [65]. Therefore, it’s worth paying attention to the TGF-β signaling pathway in PDAC and investigating the specific mechanism of tumor progression.

Some limitations should be noted. First, the regional effect on PDAC genomic landscape was not taken into account. The epidemiology study showed the prevalence of pancreatic cancer is higher in the East China region [2], but because all patients were selected in a single-center, patients were limited to a certain geographical breadth across China. Secondly, the cohort size is relatively small especially in the situation of lacking published Chinese PDAC genetic information as a reference. Thirdly, the molecular profiling were performed using a targeted panel NGS which is less comprehensive than the WES results of the TCGA database. Finally, due to the study being retrospective, patients lacked the treatment response information.

## Conclusions

The present study investigated the genetic landscape of Chinese head PDAC and compared it to the Western population. The study also provided new insights into clinicopathological features and gene alterations. A nomogram was established to predict PDAC prognosis based on tumor genetic alterations and clinical features whose performance was promising in both Chinese and Western cohorts. This study may shed light on the Chinese PDAC molecular profiling and provide a new method to predict prognosis in clinical practice.

## Supplementary Information

**Additional file 1: Table S1.** List of 425 targeted sequencing genes.

**Additional file 2: Table S2.** List of 91 TCGA patients' barcode.

**Additional file 3: Table S3.** List of all detected alterations and TMB in Chinese cohort.

**Additional file 4: Figure S1.** Survival curve of KRAS G12 subtypes, MS status, and TMB subgroups.

**Additional file 5: Figure S2.** The association between cancer antigen levels and gene alterations.

**Additional file 6: Figure S3.** Vascular invasion possibility is associated with RNF43.

**Additional file 7: Figure S4.** Gene rearrangements detected in two KRAS wild-type patients.

**Additional file 8: Table S4.** Clinical and pathological data of Chinese cohort.

**Additional file 9: Table S5.** FFPE DNA concentrations of Chinese cohort.

## Data Availability

All data generated or analyzed during this study are included in this published article and its supplementary information files.

## Abbreviations

CNV: Copy number variation
DDR: DNA damage repair
d-MMR: Mismatch repair deficiency
FFPE: Formalin-fixed, paraffin-embedded
MSI: Microsatellite instability
NGS: Next-generation sequencing
PDAC: Pancreatic ductal adenocarcinoma
SNV: Single nucleotide variant
SV: Structural variants
TMB: Tumor mutational burden
