# Supplementary figures and images for: The genetic landscape of pancreatic head ductal adenocarcinoma in China and prognosis stratification
Source: BMC Cancer. 2022 Feb 18;22:186. doi: 10.1186/s12885-022-09279-9 (PMC8855595; doi:10.1186/s12885-022-09279-9)

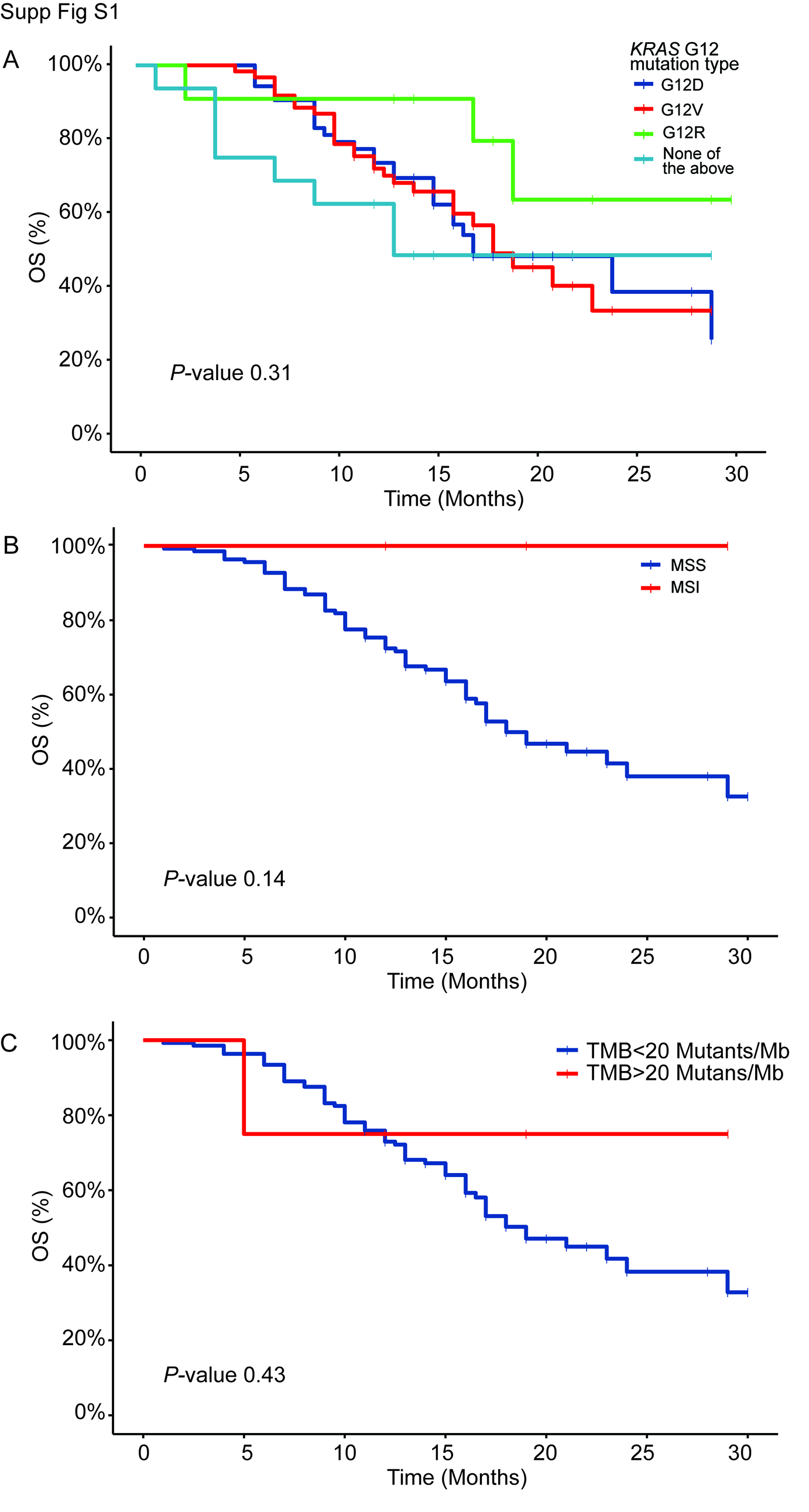

Supplement: Supplementary file 4 — Additional file 4: Figure S1. Survival curve of KRAS G12 subtypes, MS status, and TMB subgroups. [file 12885_2022_9279_MOESM4_ESM.tif]

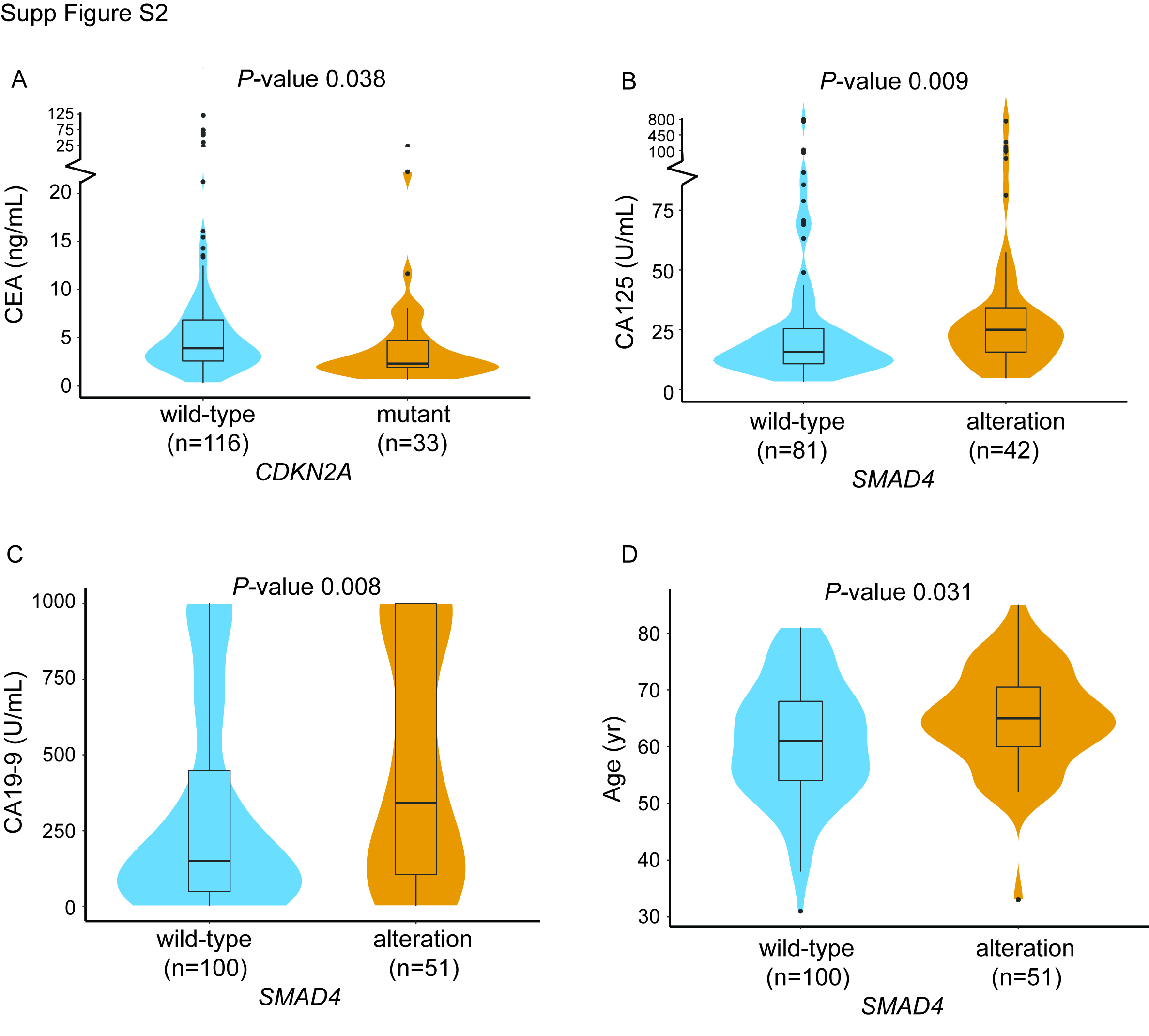

Supplement: Supplementary file 5 — Additional file 5: Figure S2. The association between cancer antigen levels and gene alterations. [file 12885_2022_9279_MOESM5_ESM.tif]

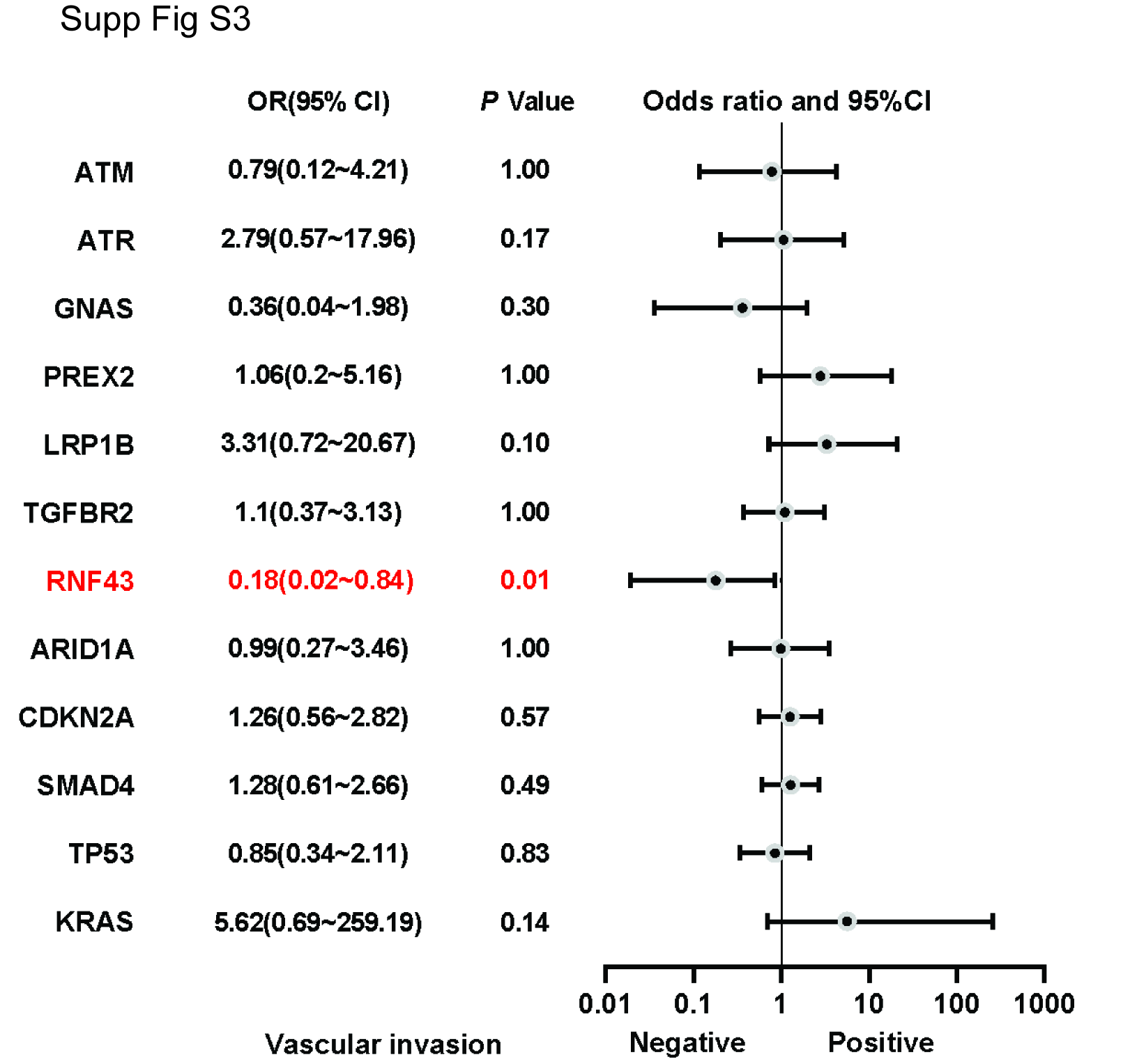

Supplement: Supplementary file 6 — Additional file 6: Figure S3. Vascular invasion possibility is associated with RNF43. [file 12885_2022_9279_MOESM6_ESM.tif]

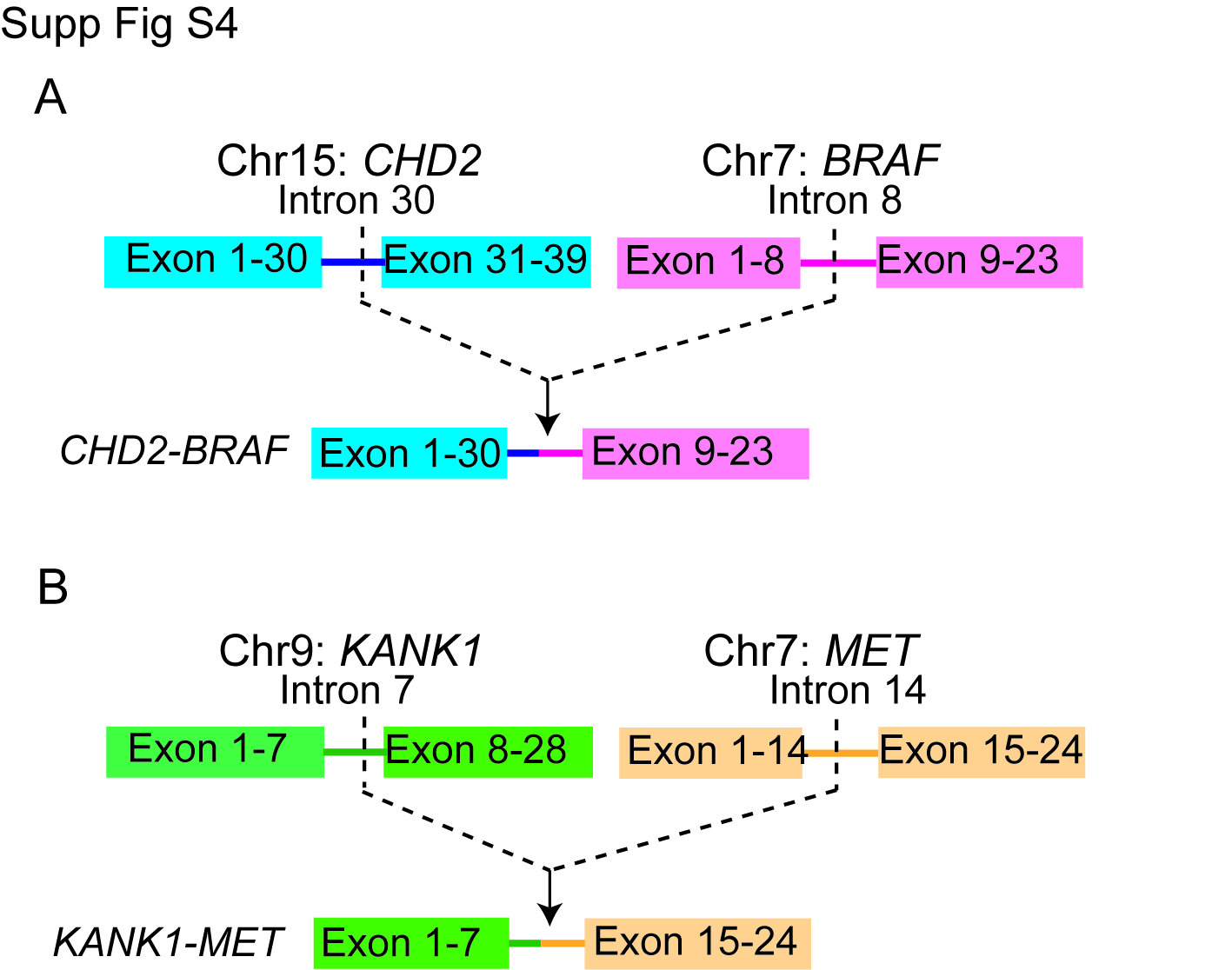

Supplement: Supplementary file 7 — Additional file 7: Figure S4. Gene rearrangements detected in two KRAS wild-type patients. [file 12885_2022_9279_MOESM7_ESM.tif]
